# Gabriel Anton (1858–1933)

**DOI:** 10.1007/s00415-021-10662-y

**Published:** 2021-06-18

**Authors:** Andrzej Grzybowski, Joanna Żołnierz

**Affiliations:** 1 Department of Ophthalmology, University of Warmia and Mazury, Olsztyn, Poland; 2 Institute for Research in Ophthalmology, Gorczyczewskiego 2/3, 61-553 Poznan, Poland; 3 Department of Humanities and Social Medicine, Medical University of Lublin, Lublin, Poland

**Fig. 1:**
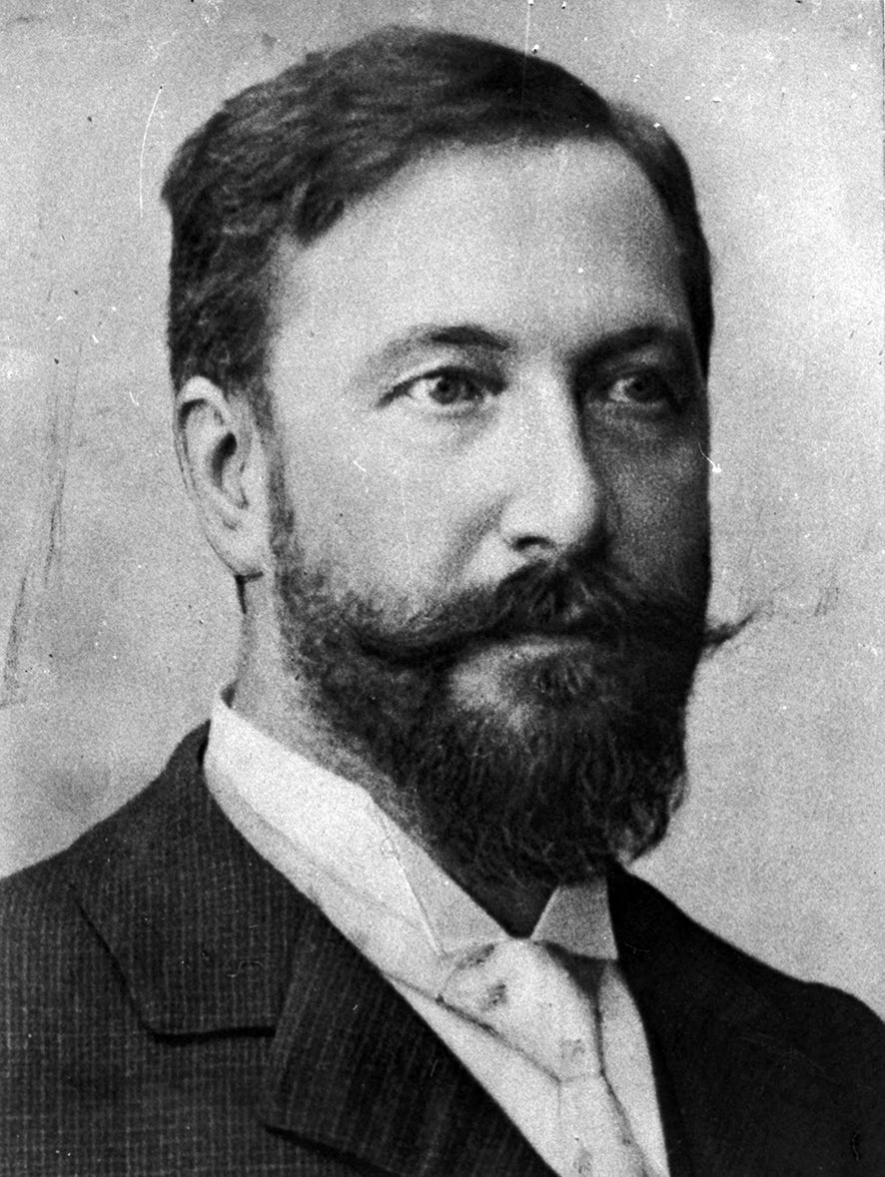
Gabriel Anton (1858–1933). *Source*: reproduced by permission of the Archive University of Graz

Gabriel Anton was born on August 28, 1858, in Saaz, Bohemia (today Žatec in the Czech Republic) (Fig. 1). After graduating in medicine from the University of Prague in 1882, Anton worked as a physiatrist and general physician in Prague and in Dobranz [1–3]. He continued his work in a doctor’s office in Prague and the hospital in Dobranz until 1887, when he began working with Theodor Hermann Meynert (1833–1892) at the Department of Neuropsychiatry in Vienna [1].

Meynert’s influence on young Anton was enormous. It is indicated that a few years under his direction marked the further course of Anton’s professional and scientific career. Excellent knowledge of the anatomy of the brain and the tendency to combine the occurrence of mental disorders with the brain’s pathomorphology, like Meynert, led even Anton’s colleagues to call him one of Meynert’s most excellent students [2]. The first teachers of Anton who directed his interests to the structure and function of the human brain were outstanding scientists: Hans Chiari (1851–1916), professor of pathological anatomy, and Arnold Pick (1851–1924), director of psychiatric institutions in Prague and Dobranz, under both of whom Anton worked as an assistant [2, 3].

Anton’s career developed at an impressive pace. At the age of 32 years, he was appointed chief of the Neuropsychiatric Hospital and Chairman of Psychiatry and Nervous Diseases at the University of Innsbruck. In 1891, 2  years after his habilitation, Anton was appointed associate professor of psychiatry and neuropathology [1, 2]. In 1893, he began researching a disorder, the description and attempted explanation of which made him a name well known to this day [1]. A work summarizing his research on anosognosia (a term introduced by Joseph Babinski only later, in 1914) was an article from 1899. In this paper, Anton presented a description of three patients who were unaware of their sensory deficiencies caused by nervous dysfunctions [4]. The first two cases—Johann F. and Juliane H.—related to bilateral damage to the temporal lobes and cortical deafness were previously published in 1898 [5]. The third case of cortical blindness (Ursula M.) was reported earlier and published in 1886 in the Communications of the Society of Physicians of Styria [1, 2].

Anton was not the first scholar who described anosognosia. Previously, this was done, among others, by Carl Wernicke in 1874, Carl Westphal in 1882, and Constantin von Monakow in 1885 [2, 6]. Nonetheless, Anton’s contribution to the description and explanation of the phenomenon of anosognosia was so crucial that on the anniversary of Anton’s 60th birthday, Albrecht proposed creating a medical eponym in his honor [2, 7].

During his stay in Innsbruck, Anton also studied the movement disturbances in chorea. When examining the case of a 9-year-old boy, he was the first to describe the role of the basal ganglia in this type of disorder [8]. It was definitely an important step in enabling further studies on extrapyramidal diseases [2, 3]. In addition, it was suggested that Anton may have been the first author to describe ‘Wilson disease’ in 1908, several years before Kinnier Wilson (1878–1937) [1].

In 1894, as a full professor, Anton began working at the University of Graz. He worked as a professor of neuropsychiatry in Graz for 11 years, until the tragic death of Carl Wernicke, after whom in 1905 Anton occupied the chair at the University of Halle, where he worked until his retirement in 1926 [1, 2]. During his stay in Halle, he conducted intensive research that was of great importance for the development of neurosurgery. Beginning in 1895, he described the nervous consequences of hydrocephalus. Anton's considerations were presented in books in 1904, and became a direct inspiration for attempts to develop effective surgical methods for lowering intracranial pressure [2]. Anton established a close collaboration initially with Fritz Gustav von Bramann (1854–1913) and subsequently with Victor Schmieden (1874–1945) and Friedrich Voelker (1872–1955), and developed new methods for decreasing intracranial pressure [2, 3]. The result of these works was the development of two surgical techniques: the ‘Balkenstich procedure’ [9] and the ‘suboccipital stitch’ [10].

Anton also worked on the issues of recovery after injuries of the central nervous system, thus contributing to the development of new techniques in neurorehabilitation. Anton recommended sensory training aimed at strengthening other functions in patients with visual impairment [2]. His scientific interests also comprised psychopathological and physical development disorders of children and adolescents. Additionally, several centers for developmentally disabled children were established [2, 3]. The first institution was founded while Anton was working in Graz, and the next public one, offering free help, was established in Halle. An observation center was also established at the University of Halle in 1916 to assess the behavior of children, who could also receive their education there. Anton also developed and described caring-educational recommendations to strengthen disturbed developmental processes in children with mental disorders. He was also a teacher for Franz Günther von Stockert (1899–1967), his future son-in-law and head of the children’s ward at a psychiatric clinic in Frankfurt am Main [2]. This activity for children and adolescents with developmental and mental disorders contrasts with Anton’s views as a supporter of the eugenic theories prevalent at the time [2, 3].

Gabriel Anton died on January 3, 1933 [3]. Appreciated for his lifetime contribution to the development of psychiatry and neurology, he was a member of many national and international scientific societies, including The German National Academy of Natural Sciences Leopoldina [2].
